# Parents’ evaluations on the service delivery and communicatıon skills of the pediatric dentists

**DOI:** 10.1093/eurpub/ckac131.329

**Published:** 2022-10-25

**Authors:** N Karamüftüoğlu, A Uğraş Dikmen, D Atabek, FS Korkmaz Öner, S Özkan

**Affiliations:** 1 Pedodontics, Dentistry Faculty, Cyprus International University, Lefkoşa, Cyprus; 2 Public Health, Gazi University, Ankara, Turkey; 3 Pedodontics, Dentistry Faculty, Gazi University, Ankara, Turkey

## Abstract

**Aim:**

In the study,it was aimed that parents who need oral health services for their children for any reason should evaluate pediatric dentists in terms of service provision and communication skills.

**Methods:**

The study was conducted with 123 parents who applied to a private oral health clinic in Ankara between 20/06/2021-20/07/2021.For parents,10 descriptive and 20 face-to-face questionnaires questioning service delivery and communication skills on a 5-point Likert scale were applied.

**Results:**

The mean age of the participants was 40.9±5.9 years.Only 34.1%(n = 42) of the parents who needed oral health services for their children directly applied to the pediatric dentist,while the others 65.9%(n = 81) applied to dentists who were not pediatric dentistry specialists.The mean score of evaluation of parents’ pediatric dentists in terms of service delivery and communication skills is 4.16±0.44.When pediatric dentists were evaluated in terms of service delivery and communication skills.74.8%(n = 92) made the explanations in a way that they could easily understand,67.8%(n = 82) did not speak fast enough to prevent them from understanding the words,66.9%(n = 79) stated that they created an environment that respects privacy and 72.6%(n = 85) stated that they allocated enough time as “always”.

**Conclusions:**

As a result, it has been determined that the frequency of parents applying to physicians who are not pediatric dentists for their children’s oral health problems is high. However,the parents who applied to the pediatric dentist found the pediatric dentist to be more competent in terms of service delivery and communication skills.It is necessary to increase the awareness of the parents about the pediatric dentistry expertise.

**Key messages:**

• Parental evaluations of pediatric dentists regarding service delivery and communication skills are important.

• Oral health literacy of parents should be developed.

